# Targeting Multiple Gut‐Brain Pathways in Obesity: Rationale for Combination Pharmacotherapy

**DOI:** 10.1002/osp4.70141

**Published:** 2026-04-04

**Authors:** Alexander D. Miras, Muzamil Hussain

**Affiliations:** ^1^ School of Medicine Ulster University Derry UK; ^2^ Investigative Medicine Imperial College London London UK

**Keywords:** GLP‐1, neuropharmacology, obesity management medication, weight management

## Abstract

**Background:**

As a disease of energy dysregulation, obesity involves metabolic, hormonal, and neural factors, the interconnection of which is referred to as the “gut‐brain axis.”

**Objective:**

This review aimed to provide an overview of the clinical evidence of physiological and objective or subjective changes in eating behavior with gut hormone analogs and NB‐ER, as well as a mechanistic rationale for the combined use of these medications to target multiple pathways along the gut‐brain axis, particularly for patients who have not achieved their health goals with a single medication.

**Findings:**

Peripheral hormones such as glucagon‐like peptide‐1 (GLP‐1) are released in response to food consumption. Peripheral signals are integrated in the hypothalamus and hindbrain to promote energy homeostasis. These brain regions also interact with other systems such as the mesolimbic dopamine system, which promotes food intake for its rewarding properties. Thus, medical interventions for obesity, such as pharmacotherapy and bariatric surgery, aim to regulate various components of this gut hormone–hedonic brain axis. Gut hormone analog medications such as liraglutide, semaglutide, and tirzepatide target the GLP‐1 receptor, with tirzepatide also targeting the glucose‐dependent insulinotropic polypeptide receptor. These gut hormone analog medications primarily exert their effects on the hypothalamus and brainstem to reduce energy intake. Evidence on their effects on the reward system and reward‐based eating is inconsistent. The fixed‐dose, extended‐release combination of naltrexone and bupropion (NB‐ER) acts via the hypothalamic and mesolimbic systems to reduce food intake and reward‐based eating.

**Conclusion:**

The distinct yet complementary effects of gut hormone analog medications and NB‐ER on gut‐brain pathways regulating satiety, hunger, and reward provide a mechanistic rationale for their combination in obesity treatment.

**Abbreviations**
BMI: body mass index
GIP: glucose‐dependent insulinotropic polypeptide
GLP‐1: glucagon‐like peptide‐1
NB‐ER: fixed‐dose, extended‐release combination of naltrexone and bupropion
POMC: pro‐opiomelanocortin

## 1 Introduction

Obesity is a complex disease of energy dysregulation that involves metabolic, hormonal, and neural factors, referred to as the “gut‐brain axis” [1, 2]. In the periphery, hormones such as glucagon‐like peptide‐1 (GLP‐1) are released in response to short‐term signals including the volume and composition of food consumed [3]. Other hormones such as leptin are released in proportion to adipose tissue stores and long‐term energy intake [3]. Energy intake and expenditure are directed by the interaction of homeostatic and hedonic brain pathways [1]. Peripheral signals from the stomach, pancreas, intestines, and adipose tissue are integrated within the hypothalamus to promote or inhibit food intake to maintain energy balance [4]. For example, pro‐opiomelanocortin (POMC)‐producing neurons in the hypothalamus are activated by anorexigenic gut hormones and adipose‐derived leptin to promote satiety [5]. Moreover, medications that target the GLP‐1 receptor also act in the hindbrain to promote satiety [6].

Homeostatic systems of the hindbrain and hypothalamus interact with one another as well as with other brain systems such as the mesolimbic dopamine system to promote the ingestion of food for its rewarding properties rather than for metabolic needs [6, 7, 8]. These systems are often dysregulated in people with obesity. For example, patients with obesity had greater hypothalamic volume and altered dopaminergic signaling compared with those without obesity [9, 10]. Moreover, in response to high‐ versus low‐calorie food images, patients with obesity and type 2 diabetes had increased activation in the hypothalamus and other brain regions compared with those without obesity [11]. Other studies showed that body mass index (BMI) positively correlated with increased activity in the caudate nucleus and right insula when anticipating chocolate milk compared with a tasteless solution [12]. Together, this suggests that patients with obesity have baseline differences in homeostatic and reward‐related neural correlates of eating behavior.

Obesity medications therefore aim to regulate various components of this gut hormone–hedonic brain axis. Gut hormone analog medications such as liraglutide, semaglutide, and tirzepatide target the GLP‐1 receptor; tirzepatide also targets the glucose‐dependent insulinotropic polypeptide (GIP) receptor [13, 14, 15]. Gut hormone analogs reduce food intake and possibly also alter the types of foods consumed [16, 17, 18, 19], potentially via effects on both the gut and brain such as delayed gastric emptying and increased satiety signaling to the brain [6, 20, 21, 22]. In clinical trials, liraglutide led to 8% body weight reduction in 56 weeks [23]. Semaglutide and tirzepatide produced more robust body weight reductions of 15%–17% in 68 weeks and 15%–21% in 72 weeks, respectively [24, 25, 26]. Other approved obesity medications are understood to act more directly on the central nervous system, such as the fixed‐dose, extended‐release combination of naltrexone and bupropion (NB‐ER), an oral obesity medication. NB‐ER increases the activity of norepinephrine and dopamine via the reuptake inhibitor bupropion to stimulate hypothalamic POMC cells and prevents the subsequent inhibition of these cells via the opioid antagonist naltrexone [27]. Moreover, the individual components of NB‐ER act on reward‐related brain regions, including the mesolimbic pathway [28, 29, 30]. Weight loss over 56 weeks for patients who completed the clinical trials of NB‐ER was between 8% and 12% for patients without type 2 diabetes and 6% for patients with type 2 diabetes [31, 32, 33, 34].

As with the management of other chronic diseases such as type 2 diabetes and hypertension [35, 36], some patients taking gut hormone analog medications may benefit from adjunctive therapy to optimize their body weight reduction. For example, in a post hoc analysis of the LIGHT cardiovascular outcomes trial, patients with type 2 diabetes who were receiving GLP‐1 receptor agonist therapy (including liraglutide and exenatide) experienced greater weight reduction with NB‐ER compared with placebo [37]. This is clinically relevant in obesity care, as approximately 9%–37% of people on gut hormone analogs (e.g., liraglutide, semaglutide, tirzepatide) did not achieve ≥ 5% body weight reduction (i.e., do not respond to treatment) in clinical trials [23, 24, 26]. Moreover, < 5% weight reduction was associated with limited improvements in obesity‐related complications [38, 39, 40, 41, 42]. Thus, health care professionals may seek to combine gut hormone analog medications and NB‐ER to better target satiety, hunger, and reward among patients with obesity.

This review sought to provide an overview of the clinical evidence of physiological and objective or subjective evidence for changes in eating behavior with these medications as well as a mechanistic rationale for the combined use of obesity medications with distinct mechanisms of action to target multiple pathways along the gut‐brain axis. While for many patients the management of concomitant type 2 diabetes or prior bariatric surgery may be additional factors to consider in a personalized treatment plan, this review focused primarily on the effects of these medications in obesity.

## 2 Obesity Medications and the Gut Hormone–Hedonic Brain Axis

### 2.1 Gut Hormone Analogs and the Gut Hormone–Hedonic Brain Axis

Gut hormone analog medications act peripherally via effects on the GLP‐1 receptor and the GIP receptor [13, 14, 15]. Endogenously, GLP‐1 is released from the small intestine and colon in response to food intake and binds to GLP‐1 receptors in diverse tissues including the pancreas, stomach, intestines, and brain [43]. In the pancreas, GLP‐1 is involved in enhancing glucose‐dependent insulin secretion, promoting the survival and proliferation of beta cells, suppressing postprandial glucagon secretion, and enhancing somatostatin release [44, 45]. Possibly through decreased glucagon and improved insulin signaling, GLP‐1 exerts indirect actions in the liver to decrease hepatic gluconeogenesis, de novo lipogenesis, and hepatic steatosis [46]. Moreover, GLP‐1 activation leads to delayed gastric emptying, reduced gastric acid secretion, and decreased intestinal motility [21, 22, 47, 48]. In adipose tissue, GLP‐1 activity improves insulin sensitivity and lipid metabolism, increases adiponectin, and decreases inflammatory cytokines [49, 50]. Notably, decreased rates of gastric emptying are associated with slower eating, decreased postprandial appetite and hunger, and increased fullness, as well as decreased caloric intake during an ad libitum meal [51, 52]. As with GLP‐1, GIP is released from the small intestine in response to food intake, and GIP receptors are distributed throughout the pancreas, adipose tissue, and brain [20, 53]; however, GIP is not believed to alter gastric emptying [22]. GIP enhances the secretion of lipoprotein lipase to promote clearance of triglycerides from circulation and prevent accumulation of ectopic fat [53].

In addition to peripheral effects, gut hormone analogs act on the brain [54, 55, 56]. Receptors for GLP‐1 are located throughout human brain tissue (e.g., cerebral cortex, hypothalamus, thalamus, caudate, putamen, globus pallidus) [20]. In a study using positron emission tomography imaging, intravenous infusion of GLP‐1 receptor agonist peptides altered glucose metabolism in the hypothalamus and brainstem compared with saline [20]. Systemically administered gut hormone analogs reach their target brain centers via 2 main pathways. These gut hormone analogs may act on circumventricular organs (areas of brain where blood‐brain barrier is more permeable) such as the postrema and median eminence and then spread to adjacent regions such as the nucleus of the solitary tract and arcuate nucleus of the hypothalamus [55, 56, 57, 58]. Another way gut hormone analogs may access the brain is via glial cells called hypothalamic tanycytes, which are found along the third ventricle and have a neuroendocrine function of detecting and transporting nutrients and hormones (e.g., insulin and GLP‐1) [55, 59, 60]. Gut hormone analogs may also activate regions guarded by the blood‐brain barrier, such as the lateral septal nucleus [55, 59, 60], reaching them by either extravasation and diffusion or assisted transport by tanycytes [55].

Despite the uncertainty of the degree of direct action that gut hormone analog medications have on the central nervous system, treatment for 17 days with the GLP‐1 receptor agonist liraglutide led to reduced activation of brain regions such as the parietal cortex, insula, and putamen in response to food images [61]. Moreover, compared with placebo, treatment with tirzepatide decreased activation in the medial frontal gyrus, cingulate gyrus, and orbitofrontal cortex in response to high‐fat and high‐sugar food cues at 6 weeks [18]. Notably, evidence suggests that altered activity in the frontal cortex, putamen, and cingulate gyrus in response to food cues may reflect changes resulting from weight reduction rather than specific GLP‐1 receptor–mediated effects. Previous research showed reductions in food cue–mediated activity in the reward‐related brain regions of the putamen, prefrontal cortex, and cingulate gyrus following bariatric surgery [62], as well as changes in the activity of the prefrontal cortex, orbitofrontal cortex, and cingulate gyrus in response to food cues following weight reductions via caloric restriction [63]. Additional research is needed to understand which effects of gut hormone analogs on the brain are mediated directly by the action of the medication on the gut‐brain axis versus by the resultant weight reduction.

### 2.2 NB‐ER and the Gut Hormone–Hedonic Brain Axis

The obesity medication NB‐ER exerts its effects via the central nervous system, especially via the hypothalamic and mesolimbic systems. NB‐ER contains bupropion, which is a norepinephrine and dopamine reuptake inhibitor that increases the activity of these neurotransmitters and may therefore stimulate hypothalamic POMC cells [27]. The other component, naltrexone, is believed to prevent the subsequent inhibition of POMC cells by their inhibitory neuropeptide beta‐endorphin [27]. Compared with placebo, NB‐ER led to increased activity in the parietal, insular, anterior cingulate, and hippocampal regions as well as decreased activity in the hypothalamus in response to food cues [64].

The individual components of NB‐ER are implicated in changes in the activity of reward‐related brain regions. For example, a crossover study of healthy male participants demonstrated that oral naltrexone monotherapy compared with placebo can increase activity in reward‐related brain regions, including the nucleus accumbens, caudate, putamen, and pallidum [28], possibly reflecting an improvement of the hypodopaminergic state observed among some patients with obesity [10]. Additionally, treatment with injectable extended‐release naltrexone, which is approved for opioid use disorder, reduced reactivity to opioid‐related cues in the nucleus accumbens and orbitofrontal cortex among patients with opioid use disorder following a detoxification program [29], which may reflect improved impulse control. Preclinical evidence suggests that bupropion treatment, which is approved for smoking cessation and depression, may increase dopamine release in the nucleus accumbens [30]. Together, these findings suggest that NB‐ER may exert its effects via actions on both hypothalamic and dopaminergic, reward‐related brain systems.

## 3 Obesity Interventions and Eating Behavior

### 3.1 Evidence for Changes in Eating Behavior With Gut Hormone Analogs

Gut hormone analog medications reduced energy consumption (typically measured in kilojoules) and improved self‐reported hunger‐ and appetite‐related measures [16, 17, 19, 65]. Among 49 patients with obesity in a randomized, controlled, crossover trial, those receiving liraglutide demonstrated a postprandial decrease in appetite and glycemia and a 16% decrease in overall energy intake versus placebo during an ad libitum lunch following 5 weeks of treatment [19]. The same study found that delayed gastric emptying with a gut hormone analog correlated with decreased appetite and early satiety [19]. Another ad libitum randomized, controlled, crossover trial of 30 patients with obesity found that semaglutide versus placebo resulted in 24% lower overall energy consumption during an inpatient stay following 12 weeks of treatment [16]. In the same trial, patients reported less hunger, better control of eating, fewer food cravings, and lower ratings of food pleasantness following a standard breakfast [16]. Similarly, patients treated with injectable semaglutide consumed 35% less energy and had a shorter meal duration during an ad libitum lunch compared with those administered placebo, as well as lower energy intake at the evening meal and evening snack in a randomized, controlled, crossover trial of 30 patients with obesity [16]. In a randomized, controlled, parallel‐group trial of 72 patients with obesity, patients treated with injectable semaglutide also consumed 35% less energy during lunch following 20 weeks of treatment, a 47% reduction in intake from baseline (versus 19% with placebo) [17]. Moreover, among 114 patients with overweight or obesity, tirzepatide treatment led to decreased caloric consumption during an ad libitum lunch compared with placebo or liraglutide treatment after 6 weeks in a randomized, controlled, parallel‐group trial [18]. In the same trial, tirzepatide versus placebo reduced overall fasting appetite, decreased fasting hunger and prospective food consumption, and increased fasting satiety and fullness as measured by self‐report using visual analog scales after 3 weeks of treatment [18]. Moreover, that trial found a reduction in overall food craving scores per the Food Craving Inventory and Food Craving Questionnaire‐State as well as perceived hunger scores per the Eating Inventory [18]. Overall, this body of evidence suggests that the delayed gastric emptying characteristic of gut hormone analog treatments, as well as possible brain‐mediated effects, results in lower overall energy consumption and changes in self‐reported hunger, satiety, and cravings.

Decreased energy consumption and improvements in cravings and fullness were also observed among patients with type 2 diabetes taking gut hormone analog medications. The GLP‐1 receptor agonist exenatide versus placebo also led to a 23% reduction in energy intake among patients with and without obesity and a 14% reduction among patients with type 2 diabetes compared with placebo [66]. In a randomized, controlled crossover trial of 15 patients with type 2 diabetes, oral semaglutide versus placebo was associated with 39% lower overall energy intake during an inpatient stay following 12 weeks of treatment [65]. Moreover, that study found that oral semaglutide decreased food cravings and improved control of eating and difficulty resisting cravings following a standard breakfast [65]. Similarly, among patients with diabetes, liraglutide led to increased fullness ratings compared with placebo [61].

There may also be differences in the types of food patients consume and prefer when taking obesity medications. For example, patients treated with semaglutide demonstrated a 35% lower intake of high‐fat and nonsweet foods during an evening snack compared with those administered placebo in the randomized, controlled, crossover trial of 30 patients with obesity, as well as decreased self‐reported preference for high‐fat and nonsweet foods and increased preference for low‐fat and sweet foods within 5 h following a standard breakfast [16]. Compared with placebo, tirzepatide decreased the fasting desire to eat sweet, salty, and fatty foods (per visual analog scale ratings) as well as cravings for high‐fat foods, sweets, carbohydrates, and fast‐food fats, without affecting cravings for fruits and vegetables on the Food Craving Inventory after 3 weeks of treatment [18]. Similarly, in the randomized, controlled, crossover trial of 15 patients with type 2 diabetes, patients treated with oral semaglutide consumed less energy from high‐fat and sweet foods than those administered placebo [65]. In contrast, the same study found no differences in the palatability of foods among patients with diabetes treated with oral semaglutide versus placebo [65]. Moreover, preclinical evidence suggests that subcutaneous tirzepatide treatment may selectively reduce the intake of high‐fat food via GLP‐1–mediated effects [67]. Additional research using objective measures of eating behavior at later time points in treatment is needed to better understand the longitudinal and food‐type specific effects of gut hormone analog medications.

The differences in specific food preferences and reported eating‐related measures observed in some studies may be due to the changes in brain activity observed following gut hormone analog treatment, such as decreased activity in reward‐related regions including the medial frontal gyrus, cingulate gyrus, and orbitofrontal cortex in response to high‐fat and high‐sugar food cues [18]. Additional research is needed to understand which specific mechanisms of gut hormone analogs along the gut hormone–hedonic brain axis alter preferences for specific food types or macronutrients and how these effects interact with the changes resulting from weight reduction.

### 3.2 Evidence for Changes in Eating Behavior With NB‐ER

In the initial phase 3 clinical trials, NB‐ER led to reductions in food cravings among patients with overweight and obesity [31, 32]. In the randomized, controlled, multicenter COR‐I trial of 1742 patients, patients reported decreased hunger, reduced frequency of cravings for starchy foods, improved control over eating, decreased difficulty resisting cravings, and reduced frequency of eating in response to cravings on the Control of Eating Questionnaire at 56 weeks with NB‐ER compared with placebo [32]. Similarly, patients reported reduced frequency of cravings and decreased difficulty in resisting food cravings per the Control of Eating Questionnaire with NB‐ER, as well as improved control over eating through 56 weeks in the randomized, controlled, multicenter COR‐II trial of 1496 patients [31]. Another open‐label study of 43 patients found that NB‐ER improved eating behavior according to the Altered Eating Behaviors Checklist among patients with obesity, even those with binge eating disorder [68]. Improvements from baseline to 16 weeks included decreased hyperphagia and social eating for patients without binge eating disorder; reduced post‐dinner eating, binge eating, and emotional eating for those with binge eating disorder; and reduced grazing and cravings for carbohydrates for both groups [68]. Moreover, a pooled post hoc analysis of patients from the 4 pivotal phase 3 trials of NB‐ER demonstrated that early improvements in control over food cravings on the Control of Eating Questionnaire led to greater weight reductions over the 56‐week trials [69].

The reduction in cravings and improved control over food intake in response to cravings with NB‐ER is likely due to increased activity of dopamine and norepinephrine. Importantly, decreased dopaminergic signaling is believed to contribute to food cravings, similar to the hypodopaminergic state underlying other reward‐related disorders [70]. Though there is a need for clinical studies examining the effects of NB‐ER on objective changes in eating behavior, preclinical evidence suggests that NB‐ER promotes an overall reduction in food intake [27, 71].

## 4 A Mechanistic Case for the Combined Use of Gut Hormone Analogs and NB‐ER

Approximately 9%–37% of people on gut hormone analogs do not achieve ≥ 5% body weight reduction [23, 24, 26], and there is a lack of clear evidence indicating which factors predict an optimal response to obesity treatments, especially among patients with obesity but without diabetes. A systematic review of obesity medications reported that while diabetes negatively predicted weight reduction, early response to treatment was a reliable positive predictor of weight reduction and maintenance [72]. A single‐center, controlled, parallel‐group trial of 136 patients with obesity reported that delayed gastric emptying at week 5 predicted > 4 kg weight reduction at week 16 with liraglutide or placebo treatment [73]. The same trial found that patients who consumed more calories during an ad libitum meal at baseline were less likely to have a > 4 kg weight reduction with liraglutide or placebo than those with lower baseline caloric intake [73]. Thus, clinical guidance is needed for the treatment of patients with a suboptimal response to gut hormone analog medications.

In patients who do not meet their weight goals with gut hormone analog medication monotherapy, targeting distinct pathways along the gut‐brain axis can potentially address distinct symptoms experienced by patients with obesity (e.g., rapid gastric emptying, food cravings, emotional eating, or impulsivity) and improve weight‐related outcomes [2]. Gut hormone analogs exert profound effects in the gut to slow gastric emptying as well as some brain‐mediated effects to increase satiety, ultimately acting as an anorectic [6, 21, 22]. Meanwhile, NB‐ER instead acts centrally on reward‐related systems and can ameliorate food cravings [27, 29, 74]. Moreover, the components of NB‐ER have demonstrated efficacy in the treatment of impulse and mood‐related disorders, including alcohol use disorder, opioid use disorder, nicotine addiction, and depression [75, 76, 77, 78]. These specific effects are supported by evidence from a pragmatic study in an obesity clinic that used the gut hormone analog medication liraglutide for patients characterized by abnormal satiety and rapid gastric emptying and NB‐ER for patients characterized by emotional hunger [74]. This phenotype‐guided treatment improved weight‐reduction outcomes, highlighting the importance of personalized obesity treatment [74]. Treatment personalization may therefore include combining medications to improve outcomes among patients with a suboptimal response to monotherapy.

**FIGURE 1:**
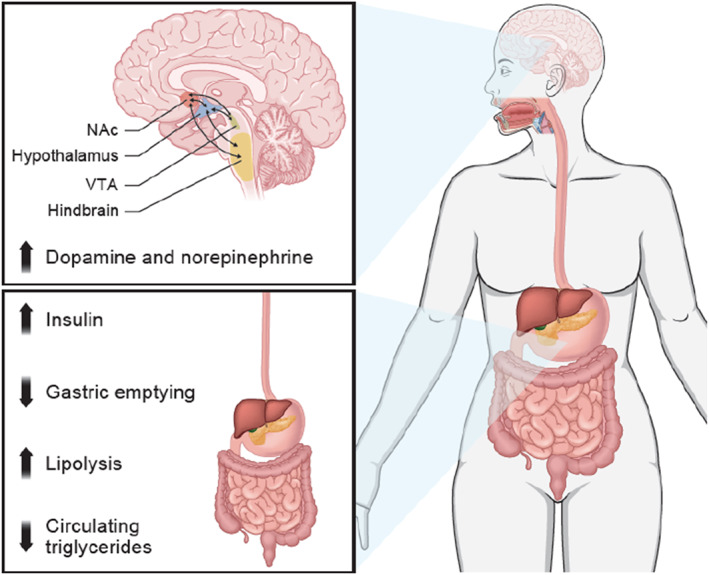
Illustration showing the complementary effects of gut hormone analogs and NB‐ER along the gut hormone–hedonic brain axis. NAc, nucleus accumbens; NB‐ER, fixed‐dose, extended‐release combination of naltrexone and bupropion; VTA, ventral tegmental area.

Patients with deficits in satiety who are using a gut hormone analog but struggle to achieve weight goals due to difficulty controlling food cravings may therefore benefit from additional treatment with NB‐ER. For these patients, the gut hormone analog therapy may address impairments in satiety [21, 22], and NB‐ER may provide additional benefits to impact reward signaling via dopaminergic mechanisms to ultimately reduce food cravings and hedonic eating [27]. Other patients who may benefit from combined treatment include those with a suboptimal response to initial treatment. In these cases, combining the peripheral effects of gut hormone analogs mediated by the GLP‐1/GIP hormone systems such as delayed gastric emptying, increased lipoprotein lipase activity [21, 22, 53], and improved glycemic control [21, 22, 43] with the central effects of NB‐ER on the dopaminergic, hindbrain, and hypothalamic systems [27] may improve weight‐related outcomes for patients with obesity (Figure 1). A post hoc analysis of patients with type 2 diabetes and overweight or obesity from the cardiovascular outcomes trial of NB‐ER found that those receiving concomitant liraglutide or exenatide experienced a 5.2% placebo‐adjusted reduction in body weight over 52 weeks when treated with NB‐ER [37]. The safety profile of combined treatment was comparable with GLP‐1 receptor agonists when used individually [37]. These findings are particularly relevant because patients with type 2 diabetes typically achieve less weight reduction with pharmacologic therapies than individuals without diabetes [79], highlighting the potential value of combining NB‐ER with gut hormone analog medications in this population. Thus, NB‐ER may be a suitable adjunct to anorectic gut hormone analog medications to promote additional weight reduction among patients who have not met their weight‐related goals with a gut hormone analog alone.

## 5 Future Directions

Randomized, controlled trials of combination treatment with the available gut hormone analog medications and NB‐ER should be conducted to further establish the safety and efficacy of the combined use of gut hormone analog medications and NB‐ER. Moreover, despite the available evidence suggesting that these medications target largely distinct yet complementary pathways to promote weight reduction in patients with obesity without additional health risks, research is needed to establish the best practices for combining multiple obesity medications in this multipronged approach. Similarly, future work should aim to identify the best indicators of a positive response to distinct obesity medications or combinations according to a patient's individual profile and factors underlying the heterogeneity in treatment response to inform personalized treatment strategies. Research should also be conducted to investigate which patients should be treated with a combination of these pharmacotherapies versus an incretin mimetic combined with phentermine‐topiramate ER or obesity medications combined with bariatric surgery to ensure each patient can find a treatment plan aligned with their individual clinical profile. In addition to a patient's specific symptoms such as cravings or hedonic eating, other factors may underlie a suboptimal response to treatment with a gut hormone analog, such as treatment adherence, improper dosing, metabolic adaptations, or genetic factors. Finally, the understanding of the gut hormone–hedonic brain axis and how obesity medications alter this axis is still evolving. Future studies should seek to reconcile the conflicting evidence about the specific neural correlates of eating behavior altered by gut hormone analog medications and the relative contribution of brain and peripheral mechanisms to the efficacy of these treatments [6, 80, 81].

## 6 Conclusions

Gut hormone analogs and NB‐ER are individually effective for obesity treatment. Gut hormone analogs may act primarily on the hypothalamus and brainstem to promote decreased energy intake, while NB‐ER may act on the central hypothalamic and dopaminergic brain systems to impact symptoms associated with obesity such as cravings. The distinct yet complementary effects of gut hormone analog medications and NB‐ER on gut‐brain pathways regulating satiety, hunger, and reward provide a mechanistic rationale for their combination in obesity treatment.

## Author Contributions

Both authors contributed to the conceptualization, writing, and editing of the manuscript.

## Funding

Development of this manuscript was sponsored by Currax Pharmaceuticals, LLC.

## Conflicts of Interest

A.D.M. reports receiving research grants from the European Union, AnaBio, Boehringer Ingelheim, Fractyl Health, Gila Therapeutics, the Jon Moulton Charitable Foundation, the Medical Research Council, the National Institute for Health and Care Research, Novo Nordisk, and Randox; receiving honoraria for lectures and presentations from Algorithm; AstraZeneca; Boehringer Ingelheim; Currax Pharmaceuticals, LLC; Eli Lilly; Ethicon; GI Dynamics; Medtronic; Novo Nordisk; and Screen Health; and being a shareholder in the Beyondbmi clinic, which provides clinical obesity care. M.H. reports receiving a research grant to his institution from Breakthrough T1D (formerly JDRF). Development of this manuscript was sponsored by Currax Pharmaceuticals, LLC.

## Data Availability

No new data are presented in this review manuscript.
